# Integrated radio-theranostics using a [^89^Zr]Zr-/[^177^Lu]Lu-labeled B7-H3 antibody-drug conjugate for prostate cancer

**DOI:** 10.7150/thno.125878

**Published:** 2026-04-16

**Authors:** Yongkang Qiu, Tingfei Gu, Tianyao Wang, Yelin Mulati, Xinyao Sun, Qi Yang, Lele Song, Tingting Yuan, Yu Fan, Lei Kang, Weibo Cai

**Affiliations:** 1 Department of Nuclear Medicine, Peking University First Hospital, Beijing 100034, China.; 2 Department of Urology, Institute of Urology, Peking University First Hospital, Peking University, The National Urological Cancer Center of China, Beijing 100034, China.; 3 Departments of Radiology and Medical Physics, University of Wisconsin - Madison, Madison, WI 53705, USA.

**Keywords:** B7-H3, Prostate cancer, Theranostics, Radioligand therapy, Antibody-drug conjugate

## Abstract

**Rationale:**

Prostate cancer remains a leading cause of cancer-related mortality in men. Although PSMA-directed theranostics have achieved clinical success, heterogeneous expression and therapy-induced downregulation limit their broad applicability. B7-H3 (CD276), which is highly and stably expressed in prostate cancer, represents a promising alternative theranostic target.

**Methods:**

A B7-H3 targeted antibody-drug conjugate (ADC) was radiolabeled with [^89^Zr]Zr- for immunoPET imaging and [^177^Lu]Lu for radionuclide therapy. *In vitro* binding specificity, *in vivo* tumor targeting, biodistribution, therapeutic efficacy, dosimetry, and safety were systematically assessed in prostate cancer xenograft models, with comparisons to radiolabeled antibody, ADC monotherapy, sequential therapy, and vehicle controls.

**Results:**

Histological analysis in prostate cancer patients suggested B7-H3 was consistently and highly expressed in primary and metastatic lesions and remained stable under therapeutic intervention. [^89^Zr]Zr-B7-H3 ADC immunoPET imaging demonstrated high and specific tumor uptake (33.2 ± 1.0 %ID/g at 144 h) and favorable tumor-to-background ratios. Therapeutic studies revealed that [^177^Lu]Lu-B7-H3 ADC achieved marked tumor growth inhibition and survival benefit, with comparable efficacy even if reduced the dose of ADC in the treatment system. Integrated [^177^Lu]Lu-ADC therapy outperformed radiolabeled antibody, ADC monotherapy, and sequential treatment strategies. No additional organ toxicity was observed compared with ADC alone, and transient hematological changes following [^177^Lu]Lu administration were reversible.

**Conclusions:**

The [^89^Zr]Zr-/[^177^Lu]Lu-B7-H3 ADC theranostic platform enables accurate imaging, precise tumor targeting, and enhanced antitumor efficacy at reduced ADC doses without increasing systemic toxicity, supporting its translational potential for prostate cancer.

## Introduction

Prostate cancer remains a leading cause of cancer-related mortality in men worldwide, with disease recurrence and metastatic progression representing the principal drivers of poor clinical outcomes. Although localized prostate cancer can often be effectively managed by surgery or radiotherapy, a substantial proportion of patients eventually develop advanced disease that requires systemic treatment 1-4. Despite recent advances in targeted therapy, immunotherapy, and radionuclide therapy, therapeutic responses in advanced prostate cancer remain heterogeneous, underscoring the need for novel targets and more effective precision treatment strategies 5-7.

Theranostics, integrating molecular imaging with targeted therapy, has emerged as a transformative paradigm in prostate cancer management, exemplified by the clinical success of PSMA-directed agents 8-10. However, PSMA expression is heterogeneous and may be downregulated during disease progression or under therapeutic pressure, limiting its universal applicability 7. These limitations have prompted the search for alternative tumor-associated targets with broader and more stable expression profiles. Among emerging candidates, B7 homolog 3 (B7-H3; CD276), an immune checkpoint molecule, has gained increasing attention due to its high and consistent expression in prostate cancer and metastases even under therapeutic intervention 11-13.

Beyond its immunomodulatory function, B7-H3 contributes to tumor growth, metastasis, angiogenesis, and therapy resistance through non-immunological mechanisms 14. These characteristics make B7-H3 an ideal target for targeted therapy and antibody-drug conjugates (ADCs), which leverage antibody specificity to deliver potent cytotoxic payloads directly to tumor cells while sparing healthy tissues 14, 15. B7-H3-targeted ADCs have demonstrated encouraging antitumor activity in prostate cancer 13; however, optimizing therapeutic efficacy while maintaining a favorable safety profile remains an important challenge 14. In this context, strategies that improve efficacy by synergistic theranostic approach and enable real-time assessment of target expression are particularly valuable.

Here, we report an integrated B7-H3-targeted theranostic platform based on a B7-H3-directed ADC, employing [^89^Zr]Zr-B7-H3 ADC for immunoPET imaging and [^177^Lu]Lu-B7-H3 ADC for targeted radionuclide therapy. This approach enables noninvasive evaluation of B7-H3 expression, precise tumor targeting, and combined cytotoxic mechanisms through radionuclide-induced DNA damage and ADC-mediated payload delivery 14, 16. Using preclinical prostate cancer models, we demonstrate that this radiation-ADC strategy achieves enhanced antitumor efficacy at reduced ADC doses without increasing systemic toxicity, supporting its potential as a clinically translatable theranostic solution for B7-H3-expressing prostate cancer.

## Materials and Methods

### Patients and tissue samples

The patients and tissue samples in this study were a subgroup of our previous study 17. Specifically, immunohistochemical (IHC) analyses were performed to evaluate the expression of prostate-specific membrane antigen (PSMA) and B7-H3 in twenty primary prostate cancer tissues and twenty bone metastasis specimens, which were randomly selected from patients treated at Peking University First Hospital between 2013 and 2023.

IHC staining intensity and antigen expression levels were quantified using an image-based semi-quantitative H-score system, consistent with the methodology described in our prior study 17. The H-score was calculated as:

score = (% weak staining × 1) + (% moderate staining × 2) + (% strong staining × 3)

Staining intensity thresholds (weak, moderate, strong) were defined according to predefined criteria within the image analysis workflow, and representative fields from each tissue section were evaluated under standardized imaging conditions.

The patients and tissue samples involved in this study were approved by the Ethics Committee of Peking University First Hospital, with exemption from informed consent (Approval No. 2023-289-001).

### Radiopharmaceutical preparation and quality control

B7-H3 ADC used in this work was generously provided by the Dept. Urology of Peking University First Hospital. The antibody moiety of the ADC is a human anti-B7-H3 monoclonal antibody (mAb) that is covalently linked to topoisomerase inhibitors, and the antibody component of this ADC is specific for human B7-H3. Another human B7-H3 mAb was used as the control, which was also provided by the Dept. Urology of Peking University First Hospital.

The affinity between B7-H3 antibody (Ab) / ADC and B7-H3 protein (KactusBio) were assessed using the Octet RED96 (Sartorius) bio-layer interferometry (BLI) system. After immersing proA biosensors in phosphate buffered saline (PBS) solution for 10 minutes, the Ab (10 µg/mL) were captured and then dipped into wells containing B7-H3 protein of 300, 150, 75, 37.5, 18.75, 9.375 and 4.688 nM for 2 minutes, with dissociation occurring in PBS solution. The affinity values were measured. Octet Analysis Studio 13.0 software (Sartorius) was used to conduct data analysis.

B7-H3 ADC was conjugated with SCN-Bn-Df (Macrocyclic) and DOTA-NHS-ester (Macrocyclic) at a molar ratio of 1:20 in a carbonate-bicarbonate buffer (pH 9.2). Purification was then performed using PD-10 columns (GE Healthcare). DFO-B7-H3 ADC was radiolabeled with [^89^Zr]Zr-oxalic acid (37-111 MBq) in 1 M oxalic acid, which was neutralized to a pH of 7.0-7.5 using 0.5 M HEPES buffer and 2 M Na_2_CO_3_, and then mixed with DFO-B7-H3 ADC at 37 °C for 2 h. For [^177^Lu]Lu labeling, DOTA-B7-H3 ADC were incubated with [^177^Lu]LuCl_3_ (300 MBq/mg), in 0.1 M sodium acetate buffer (pH 4.5-5.5) at 37 °C for 1 h. PD-10 columns (GE Healthcare) were used for purification.

The radiochemical purity of [^89^Zr]Zr-B7-H3 ADC and [^177^Lu]Lu-B7-H3 ADC was analyzed by radio thin-layer chromatography (radio-TLC), using disodium ethylene diamine tetraacetate (EDTA-2Na, 50 mM, pH 4.5) as the mobile phase and silica gel-impregnated glass fiber strips as the stationary phase.

### Western blot (WB)

Western blot analysis was performed following the standard protocols 18. Briefly, cells were collected and lysed in RIPA buffer containing 1% protease inhibitor (Beyotime Biotechnology) for 30 min on ice. After centrifugation (12,000 rpm, 4 ℃, 30 min), the supernatant was collected and protein concentration was measured using a BCA kit (Beyotime Biotechnology). Protein samples (30 μg per lane) were separated by 10% SDS-PAGE gel (120 V, 60 min) and transferred onto PVDF membranes (300 mA, 100 min). The membranes were blocked with 5% skimmed milk in TBST buffer for 2 h, then incubated with primary Ab against B7-H3 (Proteintech, 30052-1-AP, 1:8000) and β-actin (ABclonal, AC026, 1:5000) at 4 °C overnight, followed by HRP-conjugated secondary Ab (1:2000) for 1 h at room temperature. Protein bands were visualized using ChemiDoc™ MP Imaging System (Bio-Rad) with an Affinity ECL kit (ThermoFisher Scientific), and quantified via ImageJ software (v1.52, NIH).

### Cell culture and animal models

Prostate cancer cell lines (22RV1, PC-3, LNCaP, C4-2B) and control cell line (A549, lung cancer, B7-H3^-^) were purchased from the Cell Resource Center, Peking Union Medical College. All cells were cultured in RPMI 1640 medium with 10% fetal bovine serum (FBS) and 1% penicillin/streptomycin (Invitrogen) at 37 °C, 5% CO_2_.

All animal experiments were performed under protocols approved by Peking University First Hospital Animal Committee (J2024167). 6-week-old BALB/c nude male mice (Charles River) were implanted with cells at the concentration of 5 × 10^6^ cells mL^-1^ to establish subcutaneous tumor models for treatment once the tumors reached a diameter of 5-10 mm. 6-week-old male BALB/c mice (Charles River) were used for safety and toxicity evaluation.

### Flow cytometry

Following collection and washing, cells (1 × 10^6^) were centrifuged for 5 min at 500 × g. Cells were then incubated with theranostic agent B7-H3 ADC (20 μg/mL) or isotype control at 4 °C for 1 h, followed by three washes with cold PBS. Subsequently, cells were stained with a FITC-labeled anti-human IgG secondary Ab (ThermoFisher Scientific, 1:1000) at 4 °C for 1 h. After three additional washes, samples were resuspended and analyzed on a LSRFortessa cell analyzer (BD Biosciences). Data were processed using FlowJo software (v10.9, BD Life Sciences).

### PET imaging of [^89^Zr]Zr-labeled B7-H3 ADC and Ab

22RV1 and A549 tumor models were intravenously injected with [^89^Zr]Zr-B7-H3 ADC (3.0-3.7 MBq, 8-10 μg per mouse, n = 4) or [^89^Zr]Zr-B7-H3 Ab (3.0-3.7 MBq, 8-10 μg per mouse, n = 3) for PET imaging. The mice of [^89^Zr]Zr-B7-H3 ADC-block group were injected with 150 mg/kg B7-H3 ADC one day before the [^89^Zr]Zr-B7-H3 ADC injection. The mice were anesthetized with 2% isoflurane, and small animal PET/CT (Novel Medical Equipment) scans were performed at 2 h, 24 h, 48 h, 72 h, 96 h, 120 h and 144 h post injection, respectively. Quantitative results were obtained by drawing regions of interest (ROI) at tumor, heart, liver, kidney and muscle. Biodistribution was performed at 144 h post injection on a gamma counter (Hidex).

### Therapeutic administration and monitoring

Animals received [^177^Lu]Lu-B7-H3 ADC (7.4 MBq, 50 μg), [^177^Lu]Lu-B7-H3 ADC (1.9 MBq, 50 μg), [^177^Lu]Lu-B7-H3 ADC (7.4 MBq, 25 μg), [^177^Lu]Lu-B7-H3 Ab (7.4 MBq, 50 μg), [^177^Lu]Lu-IgG (7.4 MBq, 50 μg), unlabeled B7-H3 ADC (50 μg and 25 μg) or PBS vehicle respectively (n = 5 per group). In addition, a sequential therapy group was included, in which mice received unlabeled B7-H3 ADC (25 μg) on D0, followed by [^177^Lu]Lu-B7-H3 ADC (7.4 MBq, 25 μg) on D6.

Tumor dimensions and body weight were monitored over a 40-day observation period, with assessments performed every other day. Tumor dimensions were measured using calipers, and tumor volumes were calculated according to the formula: 1/2 × length × width × width. Standardized photographic documentation was obtained on D0, D6, D12, D18, D24, and D30 under consistent lighting conditions to qualitatively assess changes in tumor morphology.

Animals were monitored daily for predefined humane endpoint criteria, including tumor volume exceeding 1,000 mm^3^, or the development of severe ulceration or impaired mobility. Any animal meeting these criteria was immediately euthanized in accordance with institutional animal care guidelines.

Longitudinal SPECT/CT imaging of [^177^Lu]Lu-B7-H3 ADC (7.4 MBq, 50 μg), [^177^Lu]Lu-B7-H3 ADC (1.9 MBq, 50 μg), [^177^Lu]Lu-B7-H3 Ab (7.4 MBq, 50 μg) and [^177^Lu]Lu-IgG (7.4 MBq, 50 μg) were performed at D1, D4 and D7 post injection until the counts were too low to complete imaging.

### Safety assessments of [^177^Lu]Lu-B7-H3 ADC

Hematological analysis, biodistribution and haematoxylin and eosin (H&E) staining of major organs were performed after the treatment at D7, D14, D21 and D28 in 6-week-old, normal male BALB/c mice (n = 3, Charles River). The maximum tolerated activity was explored in 6-week-old, normal male BALB/c mice (n = 5, Charles River). Venous whole blood and serum samples were collected for whole blood cell analysis and blood biochemical tests, including alanine aminotransferase (ALT), aspartate transaminase (AST), creatinine (CREA), platelet (PLT), red blood cell (RBC), white blood cell (WBC).

### Biodistribution assessment

*Ex vivo* biodistribution studies were performed. Blood samples were collected via ocular extraction, and tumors along with major organs (heart, lung, liver, spleen, kidney, stomach, intestine, pancreas, bladder, muscle, bone and brain) were excised, weighed, and placed into counting tubes. The radioactivity of each sample was measured using a gamma counter (Hidex AMG Automatic Gamma Counter) calibrated for [^89^Zr]Zr- or [^177^Lu]Lu.

### Histological staining

Hematoxylin-eosin (H&E) staining was performed on tumor, heart, liver, spleen, kidney and stomach of mice according to a standard procedure 19. For immunohistochemical (IHC) analysis, tumor sections were used to evaluate the expression of Ki-67 and B7-H3. Briefly, 5-μm-thick serial sections were dewaxed, rehydrated, and subjected to antigen retrieval. Sections were then incubated overnight at 4 °C with primary Ab against B7-H3 (Abcam, ab227670, 1:100) or Ki-67 (Servicebio, GB111499, 1:500). After washing, corresponding HRP-conjugated secondary Ab were applied for 1 h at room temperature. Signals were developed using DAB, and sections were counterstained with hematoxylin. All images were acquired using an A1R confocal microscope (Nikon).

### Statistical analysis

Statistical analyses were performed using R (version 4.4.1). Continuous variables were expressed as mean ± standard deviation (SD). Two-way analysis of variance (ANOVA) and Student's t-test were used for comparisons among groups, with Pearson correlation and Spearman correlation for association analyses. *P* < 0.05 was considered statistically significant.

## Results

### Clinical characterization of B7-H3 expression

Baseline characteristics of patients in clinical specimen analysis could be seen in **Table S1**, including median age (66.5 years, IQR 64.8-73.3), t-PSA levels (23.4 ng/mL, IQR 12.7-79.4), and International Society of Urological Pathology (ISUP) grade distribution (primary tumors: Grade 1-5). Bone metastases were characterized by AR expression (Negative: 20%, Weak: 10%, Moderate: 30%, Strong: 40%).

**Figure 1:**
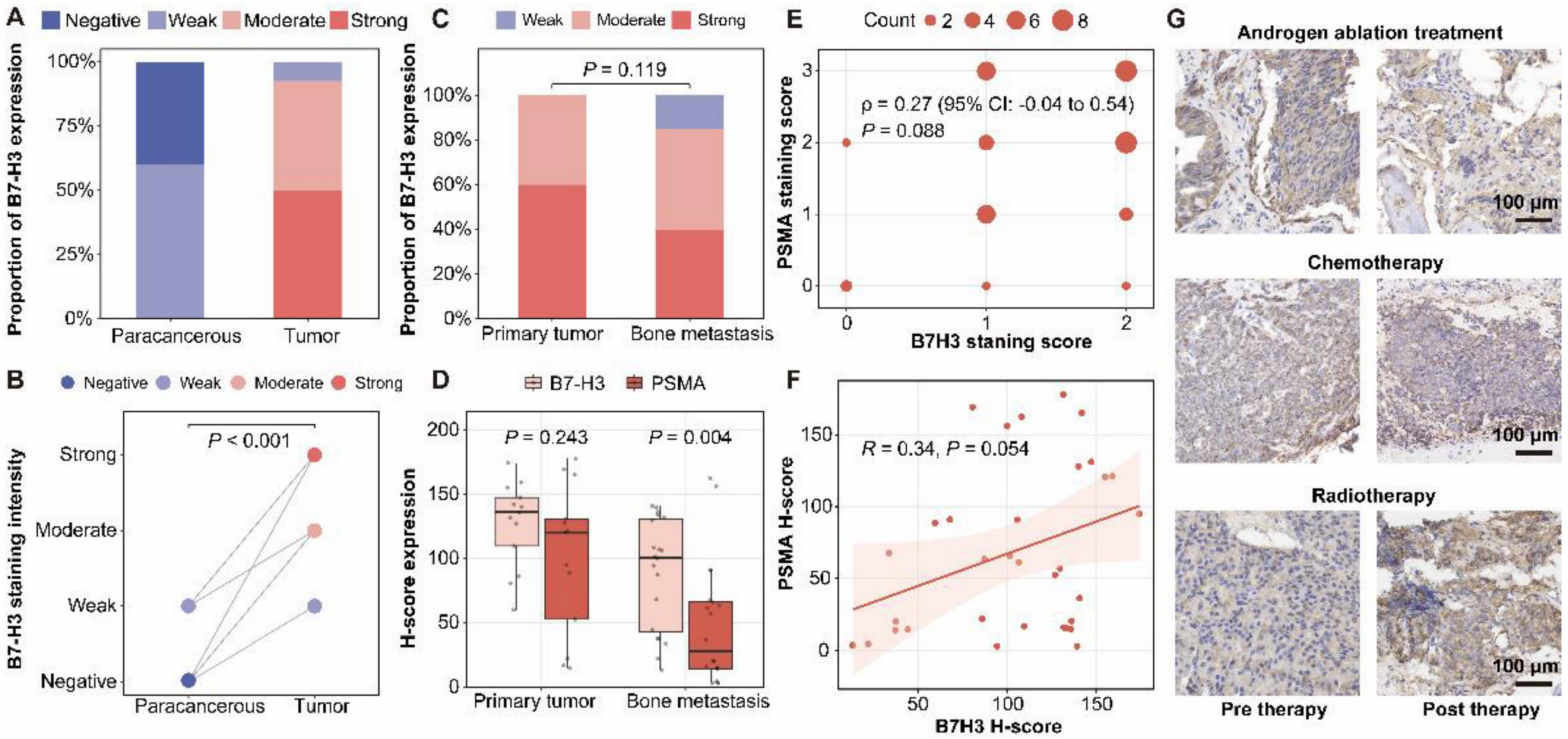
** B7-H3 is highly expressed in prostate cancer and bone metastases, and remains stable under therapeutic intervention. (A)** Distribution of B7-H3 immunohistochemical staining grades in paired para-cancerous and tumor tissues. **(B)** Comparative analysis of B7-H3 staining intensity between para-cancerous and tumor tissues. **(C)** Distribution of B7-H3 staining grades in primary tumors vs. bone metastasis specimens. **(D)** Quantitative comparison of H-scores for B7-H3 and PSMA expression in primary tumors and bone metastases. **(E)** Spearman's rank correlation between PSMA and B7-H3 staining scores. **(F)** Pearson correlation analysis between PSMA and B7-H3 H-scores. **(G)** Representative B7-H3 immunohistochemical staining in tumor tissues before and after therapy: androgen ablation therapy (top); chemotherapy (middle); radiotherapy (bottom).

Immunohistochemical analysis of B7-H3 expression patterns in prostate cancer patient-derived samples revealed significantly higher staining intensity in tumor tissues compared to matched para-cancerous tissues (*P* < 0.001, **Figure 1A-B**). Comparative analysis of B7-H3 expression patterns revealed no statistically significant difference between primary tumors and bone metastases (*P* = 0.119) (**Figure 1C**). The H-score of B7-H3 staining intensity was higher in primary tumors compared with bone metastases (**Figure S1A**), but in the strong staining subgroup, no statistically significant difference was observed between primary tumors and bone metastatic lesions (**Figure S1B**). While primary tumors and bone metastases exhibited comparable B7-H3 levels, B7-H3 expression was consistently high in both primary and metastatic lesions, with significantly higher retention in metastases than PSMA (*P* = 0.004), indicating stable expression across prostate cancer (**Figure 1D**). Correlation analyses revealed no significant association between B7-H3 and PSMA expression levels, as evidenced by both Spearman (ρ = 0.27, *P* = 0.088, **Figure 1E**) and Pearson (*R* = 0.34, *P* = 0.054, **Figure 1F**) correlation coefficients. Therapeutic intervention monitoring showed limited efficacy of conventional treatments in modulating B7-H3 expression, with androgen ablation therapy, chemotherapy, and radiotherapy all demonstrating minimal impact on B7-H3 levels in treated tumor tissues (**Figure 1G**).

### Construction and *in vitro* validation of a B7-H3-targeted theranostic ADC

**Figure 2:**
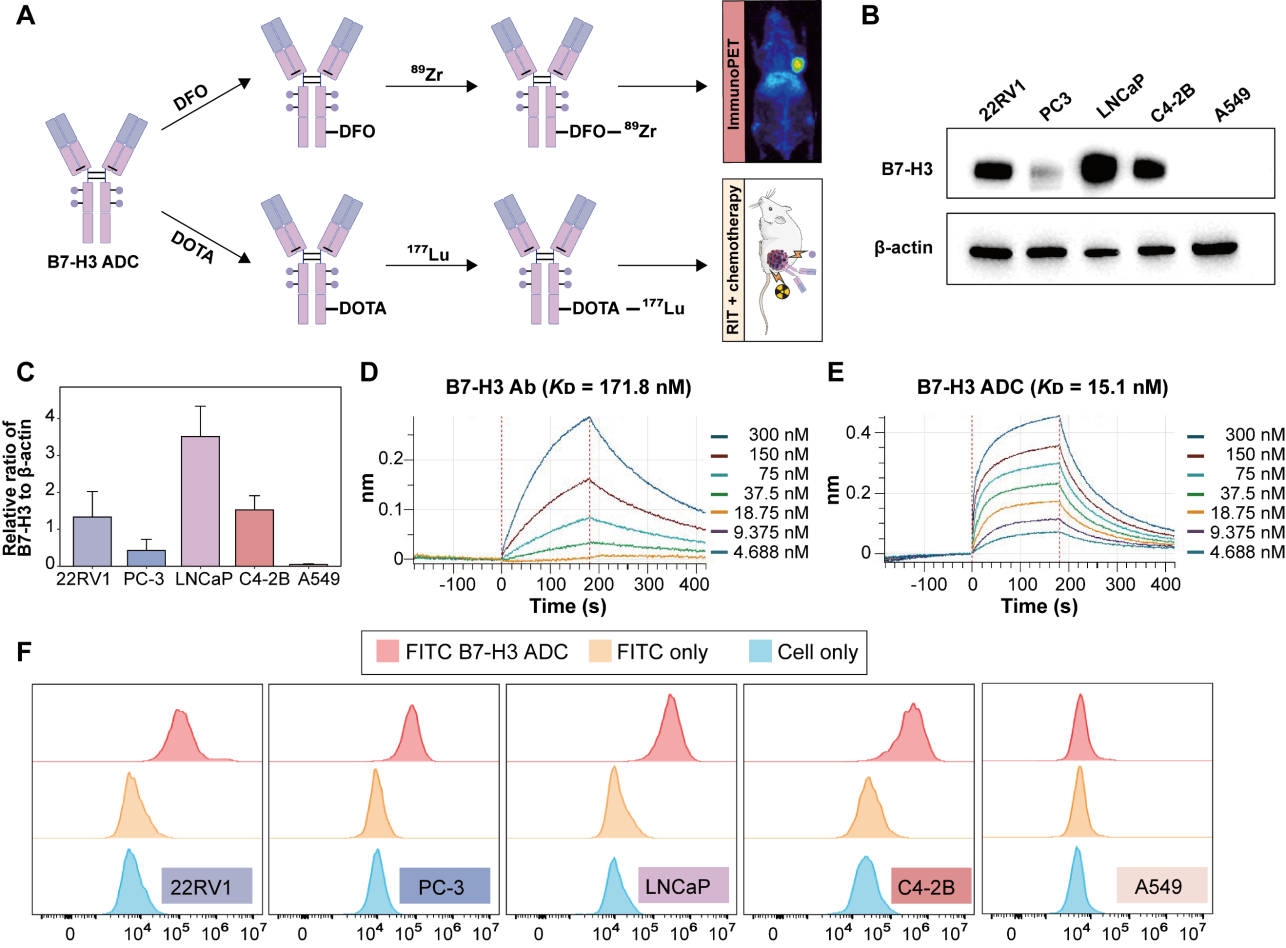
** Theranostic design of radiolabeled B7-H3 ADC and *in vitro* evaluation. (A)** Scheme illustration of the theranostic role of [^89^Zr]Zr- and [^177^Lu]Lu-labeled B7-H3 ADC for imaging and radiotherapy. **(B)** WB analysis of B7-H3 protein expression in prostate cancer cell lines (22RV1, PC-3, LNCaP, C4-2B) and negative control A549 cell line. β-actin was used as an internal reference. **(C)** Relative densitometric analysis of B7-H3 protein expression (normalized to β-actin). **(D)** Bio-layer interferometry (BLI) analysis of B7-H3 Ab to B7-H3 protein. **(E)** Bio-layer interferometry (BLI) analysis of B7-H3 ADC to B7-H3 protein. **(F)** Flow cytometry analysis confirming specific binding of B7-H3 ADC to prostate cancer cell lines (22RV1, PC-3, LNCaP, C4-2B).

As outlined in **Figure 2A**, [^89^Zr]Zr- and [^177^Lu]Lu labeled B7-H3 ADC were prepared by conjugating DFO and DOTA to explore the potential theranostic application of B7-H3 ADC. The labeling yield of [^177^Lu]Lu-labeled B7-H3 ADC was more than 60% and the radiochemical purity was more than 99%. The specific activity of the final [^177^Lu]Lu labeled B7-H3 ADC was approximately 370 MBq/mg. [^177^Lu]Lu-labeled ADC could maintain good stability regardless of whether it was stored in 0.01 M PBS and 5% human serum albumin (HSA) (**Figure S2**).

Western blot analysis demonstrated high B7-H3 expression in both androgen-sensitive (LNCaP, 22RV1) and castration-resistant (PC-3, C4-2B) prostate cancer cell lines, compared to control A549 cells, which showed negligible expression (**Figure 2B-C**). BLI analysis indicated that the B7-H3 ADC exhibited stronger binding affinity to B7-H3 protein compared with the B7-H3 Ab, with KD values of 15.1 nM and 171.8 nM, respectively (**Figure 2D-E**). Flow cytometry further confirmed specific binding of the B7-H3 ADC to B7-H3-positive prostate cancer cell lines (**Figure 2F**).

### ImmunoPET imaging and biodistribution of [^89^Zr]Zr-B7-H3 ADC

**Figure 3:**
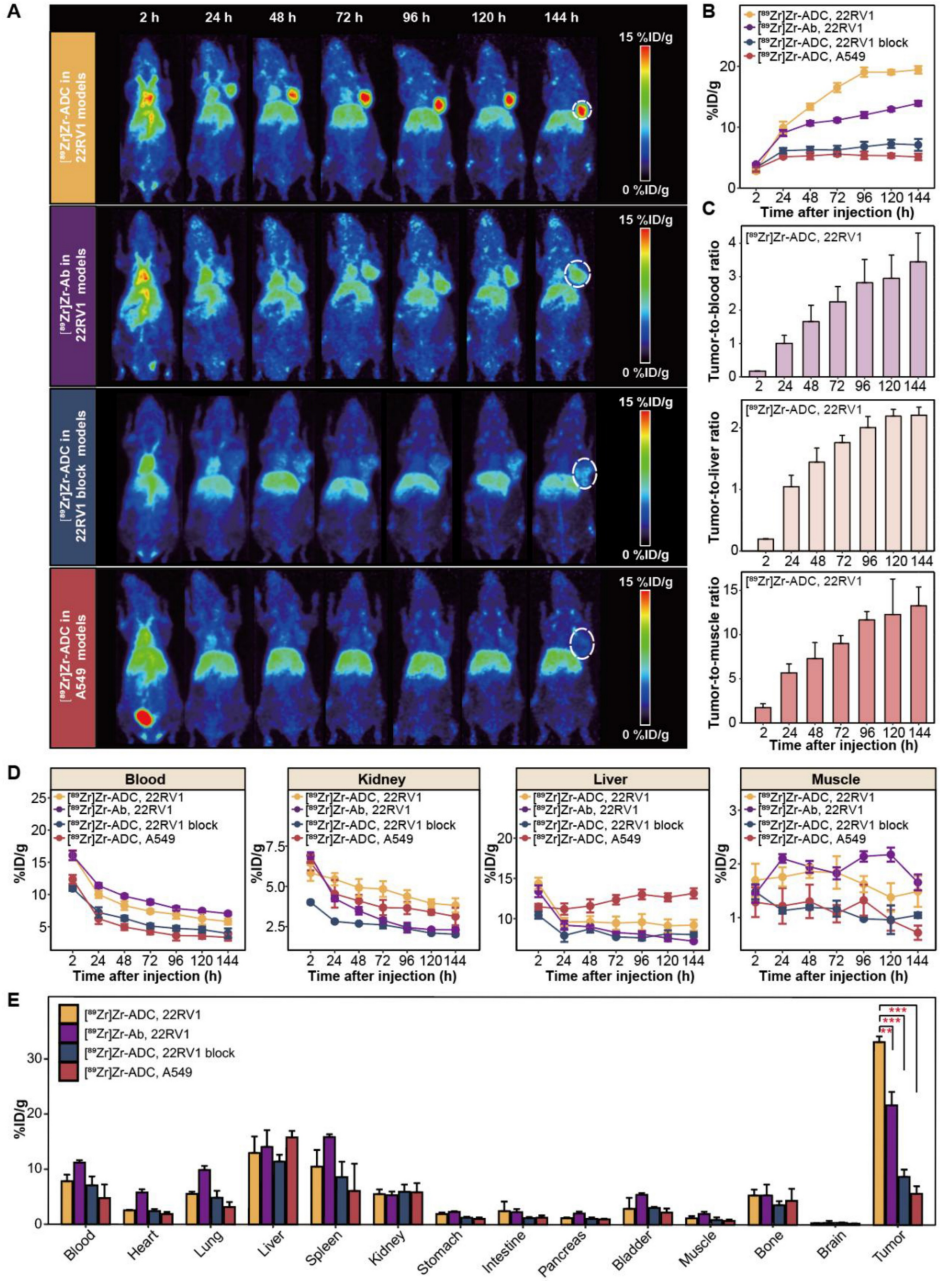
** ImmunoPET imaging and biodistribution reveals specific and sustained tumor uptake of ⁸⁹Zr-B7-H3 ADC *in vivo*. (A)** Maximum intensity projection (MIP) PET images demonstrated time-dependent tumor uptake of [^89^Zr]Zr-B7-H3 ADC in B7-H3-positive 22RV1 tumor (circle), with minimal uptake in 22RV1 block and A549 tumors. [^89^Zr]Zr-B7-H3 Ab showed lower tumor uptake than [^89^Zr]Zr-B7-H3 ADC. **(B)** Quantitative Region of Interest (ROI) analysis (n = 4). **(C)** Time-activity curves of [^89^Zr]Zr-B7-H3 ADC in 22RV1 tumor models showing tumor-to-background ratios (TBRs) for blood (top), liver (middle), and muscle (bottom, n = 4). **(D)** Time-activity curves of [^89^Zr]Zr-B7-H3 ADC in 22RV1 tumor models showing off-target organ uptake in blood, kidney, liver and muscle (n = 4). **(E)** *Ex vivo* biodistribution at 144 h confirmed specific tumor targeting (n = 4).

22RV1, 22RV1 block and A549 models were established for PET imaging with [^89^Zr]Zr-B7-H3 ADC. Serial maximum intensity projection (MIP) PET/CT images demonstrated prominent [^89^Zr]Zr-B7-H3 ADC accumulation in B7-H3-positive 22RV1 tumors, whereas markedly reduced uptake was observed in both the 22RV1 blocking group and A549 tumors (**Figure 3A**). Compared with [^89^Zr]Zr-B7-H3 Ab, immunoPET imaging with [^89^Zr]Zr-B7-H3 ADC revealed higher specificity and time-dependent targeting of B7-H3-expressing tumors *in vivo* (**Figure 3A**). Quantitative ROI analysis further confirmed significantly higher tumor uptake of [^89^Zr]Zr-B7-H3 ADC in 22RV1 tumors compared with [^89^Zr]Zr-B7-H3 Ab in 22RV1 tumors, as well as [^89^Zr]Zr-B7-H3 ADC in 22RV1 blocking and A549 models (*P* < 0.001; **Figure 3B**). Time-activity curve analysis of [^89^Zr]Zr-B7-H3 ADC in 22RV1 tumors showed progressively increasing tumor-to-background ratios relative to blood, muscle, and liver over the imaging period from 2 to 144 h post-injection (**Figure 3C**). Analysis of off-target uptake revealed initially high accumulation of [^89^Zr]Zr-B7-H3 ADC in the liver and blood, with uptake values of 14.4 ± 1.2 and 16.1 ± 1.3 %ID/g at 2 h post-injection, respectively. Over time, [^89^Zr]Zr-B7-H3 ADC exhibited progressive clearance from blood, muscle, and kidney, whereas hepatic retention remained relatively stable. By 144 h post-injection, residual blood activity decreased to 5.8 ± 1.1 %ID/g, while liver uptake persisted at 9.2 ± 1.7 %ID/g (**Figure 3D**). *Ex vivo* biodistribution analysis at 144 h post-injection corroborated the *in vivo* imaging results, demonstrating significantly higher tumor uptake in [^89^Zr]Zr-B7-H3 ADC-treated 22RV1 models (33.2 ± 1.0 %ID/g) compared with [^89^Zr]Zr-B7-H3 Ab-treated 22RV1 models (21.7 ± 2.4 %ID/g; *P* = 0.008), 22RV1 blocking models (8.8 ± 1.3 %ID/g; *P* < 0.001), and A549 models (5.7 ± 1.4 %ID/g; *P* < 0.001, **Figure 3E**).

### [^177^Lu]Lu-B7-H3 ADC achieves superior tumor growth inhibition

Treatment with [^177^Lu]Lu-B7-H3 ADC resulted in significant and radiation dose-dependent inhibition of tumor growth in 22RV1 tumor-bearing mice. Representative tumor photographs revealed pronounced tumor growth suppression in mice treated with [^177^Lu]Lu-B7-H3 ADC at 7.4 MBq, with comparable efficacy observed between the 50 μg and 25 μg ADC dose groups (**Figure S3**), and the low radiation activity group (1.9 MBq, 50 μg) and [^177^Lu]Lu-IgG (7.4 MBq, 50 μg) also showed tumor inhibition effect (**Figure S4, S5**). [^177^Lu]Lu-ADC groups demonstrated stronger tumor inhibition than [^177^Lu]Lu-Ab (7.4 MBq, 50 μg), ADC monotherapy (50 μg and 25 μg), and PBS controls. Longitudinal tumor growth curves quantitatively confirmed this hierarchy of therapeutic efficacy: [^177^Lu]Lu-ADC (7.4 MBq, 50 μg) ≈ [^177^Lu]Lu-ADC (7.4 MBq, 25 μg) > ADC (25 μg) + [^177^Lu]Lu-ADC (7.4 MBq, 25 μg) sequential therapy > [^177^Lu]Lu-Ab (7.4 MBq, 50 μg) > ADC (50 μg) > ADC (25 μg) > PBS (**Figure S3**).

**Figure 4:**
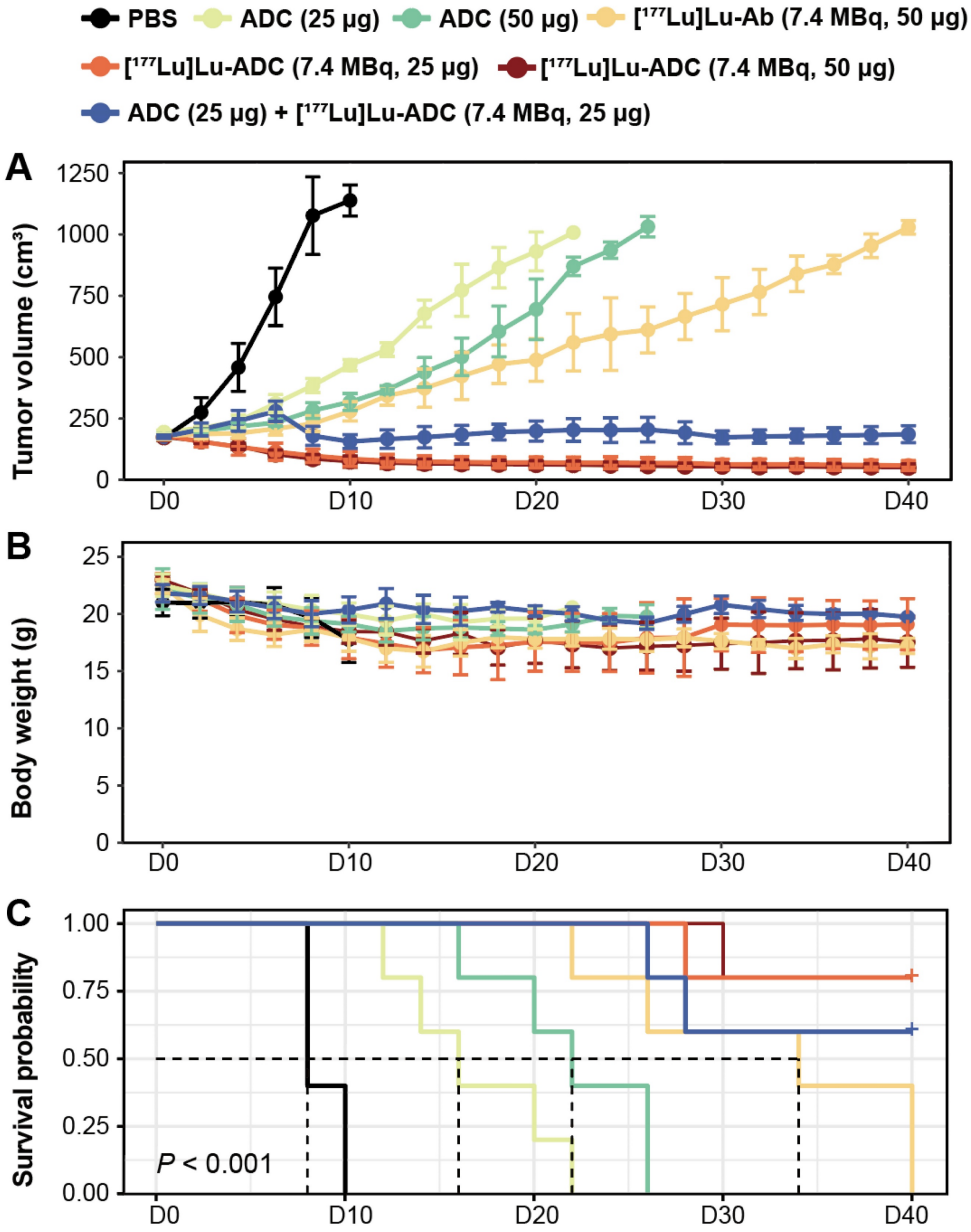
** [^177^Lu]Lu-B7-H3 ADC produces superior tumor growth inhibition compared with radiolabeled Ab and ADC monotherapy. (A)** Tumor growth curves of different treatment groups confirmed the visual observations (n = 5). Overall therapeutic efficacy followed the order: [^177^Lu]Lu-ADC (7.4 MBq, 50 μg) ≈ [^177^Lu]Lu-ADC (7.4 MBq, 25 μg) > sequential therapy > [^177^Lu]Lu-Ab (7.4 MBq, 50 μg) > ADC (50 μg) > ADC (25 μg) > PBS. **(B)** Body weight monitoring showed no significant treatment-related weight loss, indicating good tolerability (n = 5). **(C)** Kaplan-Meier survival analysis demonstrated a clear survival benefit in mice treated with [^177^Lu]Lu-ADC (n = 5).

Body weight monitoring showed no significant treatment-related weight loss across groups (**Figure 4B**). Kaplan-Meier analysis demonstrated a clear survival benefit in mice treated with [^177^Lu]Lu-B7-H3 ADC (**Figure 4C**). Individual tumor growth trajectories further highlighted the consistency of therapeutic responses in the [^177^Lu]Lu-ADC groups (**Figure S3**).

### SPECT/CT and histological analyses confirm enhanced therapeutic efficacy

**Figure 5:**
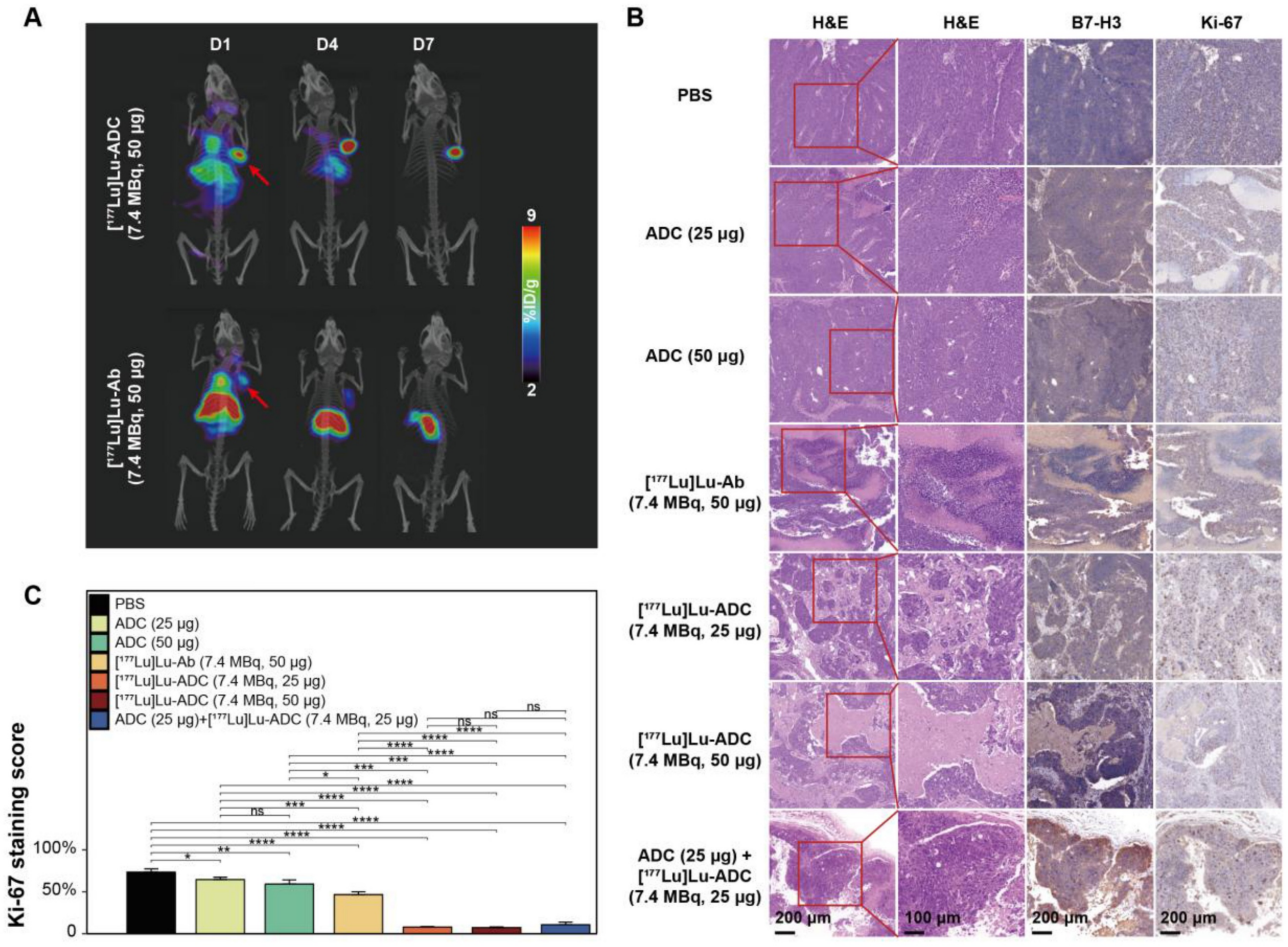
** Enhanced therapeutic efficacy of [^177^Lu]Lu-B7-H3 ADC is confirmed by molecular and histopathological analyses. (A)** Serial SPECT/CT maximum intensity projection (MIP) images of [^177^Lu]Lu-B7-H3 ADC (7.4 MBq, 50 μg) vs. [^177^Lu]Lu-Ab (7.4 MBq, 50 μg) in 22RV1 tumor-bearing mice at D1, D4, D7 post injection (red arrows indicate tumor foci). **(B)** Representative H&E staining demonstrating distinct therapeutic responses across treatment groups. B7-H3 immunohistochemistry confirmed high target expression across all groups. Ki-67 staining demonstrated different suppression across treatment groups. **(C)** Ki-67 proliferation analysis revealed significant suppression in the [^177^Lu]Lu-B7-H3 ADC group compared to other groups (n = 5).

To further validate therapeutic outcomes, SPECT/CT imaging and histopathological analyses were performed. Serial SPECT/CT imaging demonstrated persistent tumor accumulation of [^177^Lu]Lu-B7-H3 ADC, whereas tumor uptake of [^177^Lu]Lu-Ab was comparatively limited (**Figure 5A**). Histological examination revealed extensive tumor damage in the [^177^Lu]Lu-B7-H3 ADC-treated group. B7-H3 immunohistochemistry confirmed sustained target expression across all treatment groups, while Ki-67 staining showed marked suppression of tumor cell proliferation following [^177^Lu]Lu-B7-H3 ADC treatment (**Figure 5B**). Quantitative analysis confirmed significantly lower Ki-67 indices in the [^177^Lu]Lu-ADC group compared with all other groups: [^177^Lu]Lu-ADC (7.4 MBq, 50 μg) ≈ [^177^Lu]Lu-ADC (7.4 MBq, 25 μg) < ADC (25 μg) + [^177^Lu]Lu-ADC (7.4 MBq, 25 μg) sequential therapy < [^177^Lu]Lu-Ab (7.4 MBq, 50 μg) < ADC (50 μg) < ADC (25 μg) < PBS (**Figure 5C**).

### [^177^Lu]Lu-B7-H3 ADC demonstrates acceptable safety and dosimetry profiles

**Figure 6:**
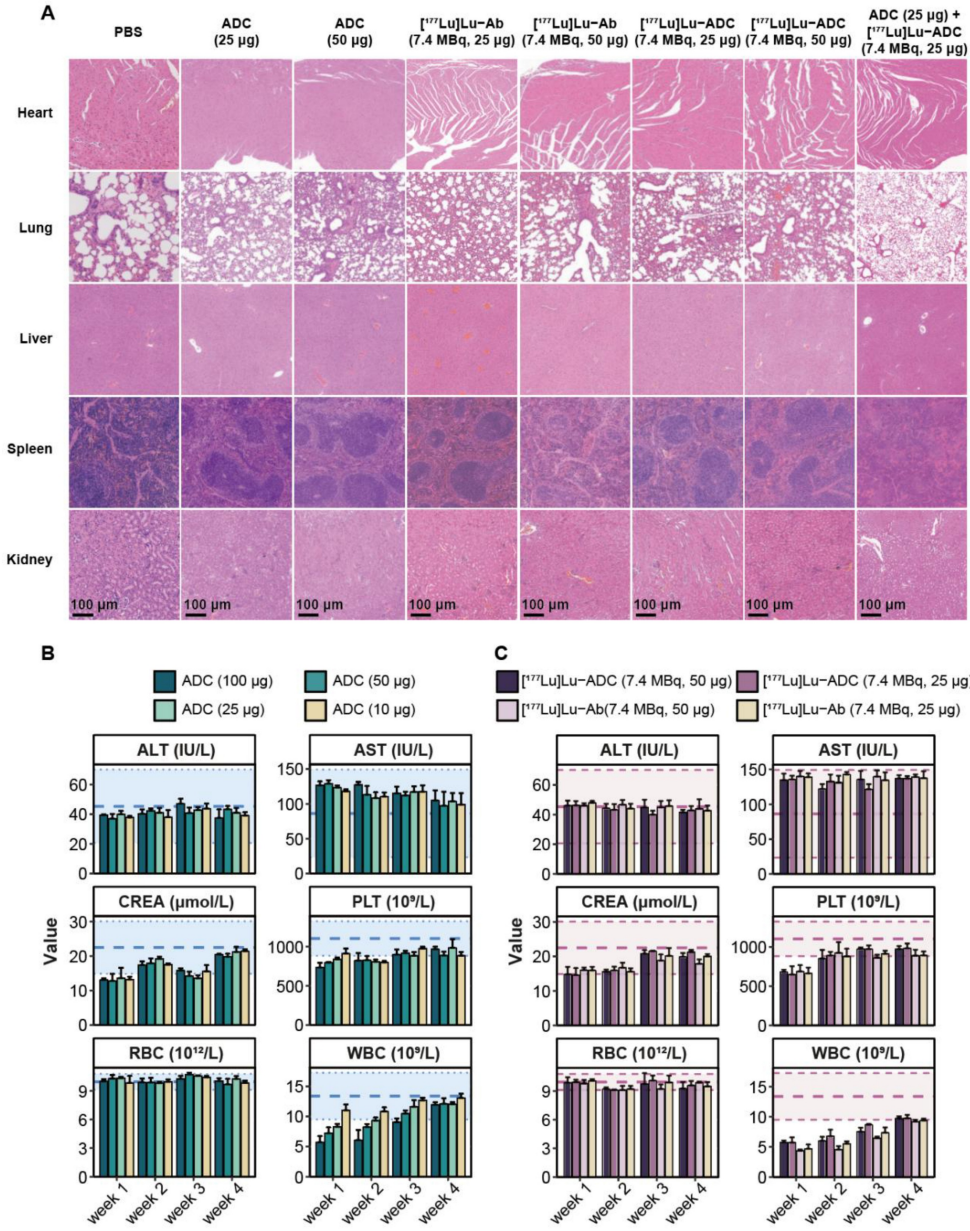
** [^177^Lu]Lu-B7-H3 ADC exhibits acceptable safety with no additional organ toxicity compared with B7-H3 ADC. (A)** Histopathological evaluation of major organs at the end of treatment or D40 post-treatment, including heart, lung, liver, spleen, and kidney, showed no overt treatment-related pathological abnormalities in any group. **(B)** Longitudinal hematological analysis revealed transient alterations in white blood cell counts in mice treated with different ADC groups (n = 3). **(C)** Longitudinal hematological analysis revealed transient alterations in white blood cell counts in mice treated with [^177^Lu]Lu-ADC and [^177^Lu]Lu-Ab (n = 3).

Safety evaluation revealed no significant histopathological abnormalities in major organs, including the heart, lung, liver, spleen, and kidney, at the end of treatment or D40 post-treatment across all groups (**Figure 6A**). Longitudinal hematological analysis showed transient reductions in white blood cell counts in mice treated with ADC-containing regimens (**Figure 6B**). Although transient leukopenia was observed in both [^177^Lu]Lu-B7-H3 ADC and [^177^Lu]Lu-Ab treated mice, white blood cell counts gradually recovered over time (**Figure 6C**). Consistent safety was also observed at [^177^Lu]Lu-IgG (7.4 MBq, 50 μg) and [^177^Lu]Lu-B7-H3 ADC (1.9 MBq, 50 μg) group (**Figure S4, S5**). In healthy BALB/c mice, the biodistribution results of different activity of [^177^Lu]Lu-B7-H3 ADC (7.4 MBq, 50 μg and 1.9 MBq, 50 μg) were similar (**Figure S4**). Preliminary maximal tolerance activity of [^177^Lu]Lu-B7-H3 ADC was explored in normal male BALB/c mice (**Figure S6**), and the potentially tolerated activity in mice may be greater than 18.5 MBq, 100 μg.

## Discussion

Theranostics represents an evolving paradigm in precision oncology, integrating diagnostic and therapeutic functions through matched radioactive isotopes 20-22. This approach has gained particular application in prostate cancer management, where PSMA-targeted agents have demonstrated both clinical and commercial success 23; however, heterogeneous expression, adaptive downregulation under therapeutic pressure, and limited applicability in certain disease states underscore the need for alternative molecular targets and more robust theranostic architectures 24-26. In this context, the present study establishes a B7-H3-targeted Zr/Lu-ADC theranostic platform that integrates target evaluation, patient stratification, and synergistic therapy at the level of a single molecular construct.

B7-H3 represents a particularly attractive target for prostate cancer theranostics 12. Unlike immune checkpoints regulated predominantly by interferon signaling, B7-H3 expression is closely linked to androgen receptor activity 27, resulting in consistently high expression across hormone-sensitive and castration-resistant prostate cancer 17, 28. This stability, together with its association with aggressive disease features, positions B7-H3 as an attractive candidate for both imaging and therapy 29. Importantly, our clinical data demonstrate that B7-H3 expression is maintained following standard therapeutic interventions, supporting its suitability for longitudinal monitoring and repeated targeting. Although primary tumors exhibited higher mean H-scores, no statistically significant difference was observed in the categorical distribution of staining intensities or within the strong staining subgroup, indicating broadly comparable B7-H3 expression patterns across disease sites, and such a distribution profile supports the potential utility of B7-H3-targeted imaging and therapy across different stages of disease.

A key conceptual advance of this study lies in the construction of a Zr/Lu-ADC-based theranostic platform. Previous B7-H3-targeted PET probes have largely relied on radiolabeled Ab or affibody-based constructs, which have shown promising tumor targeting in several solid tumors but have not been explored in prostate cancer 30, 31. In our study, direct comparison revealed that [^89^Zr]Zr-labeled B7-H3 Ab exhibited inferior tumor retention relative to [^89^Zr]Zr-B7-H3 ADC. Consequently, we selected [^89^Zr]Zr-B7-H3 ADC—not [^89^Zr]Zr-B7-H3 Ab—as the imaging agent, ensuring that diagnostic imaging faithfully reflects the *in vivo* behavior of the therapeutic construct. This design choice establishes a coherent theranostic paradigm in which [^89^Zr]Zr-B7-H3 ADC serves as a companion imaging agent for [^177^Lu]Lu-B7-H3 ADC therapy, enabling accurate patient selection, lesion visualization, and dosimetric planning 32.

Beyond imaging, therapeutic targeting of B7-H3 continues to evolve. Monoclonal Ab such as enoblituzumab have demonstrated acceptable safety profiles in early clinical settings 13, whereas B7-H3-directed ADCs, including vobramitamab duocarmazine (MGC018) and ifinatamab deruxtecan (DS-7300), have shown meaningful antitumor activity in metastatic castration-resistant prostate cancer. Nonetheless, clinical experience has highlighted a narrow therapeutic window, with hematologic toxicities remaining a dose-limiting factor, including grade ≥ 3 neutropenia (25-32%) and anemia (~25%) 14, 33, 34. These observations emphasize the need for strategies that enhance antitumor efficacy without proportionally increasing systemic exposure.

Radioimmunotherapy offers a rational solution by combining targeted radiation delivery with molecular specificity. Preclinical studies targeting B7-H3 with α- and β-emitting radionuclides have demonstrated substantial tumor control across multiple solid tumor models 35, 36. Building upon this foundation, our results show that [^177^Lu]Lu-B7-H3 ADC induces robust tumor growth inhibition in prostate cancer models following a single administration. Notably, comparable therapeutic efficacy was achieved at reduced ADC doses, indicating that the observed antitumor effect cannot be attributed solely to the radionuclide or the ADC component alone. Instead, these results support the presence of a true therapeutic synergy arising from the integrated delivery of radiation and cytotoxic payload within a single molecular entity.

Importantly, we further interrogated this synergy by evaluating a sequential treatment strategy, in which unlabeled B7-H3 ADC was administered prior to [^177^Lu]Lu-B7-H3 ADC. This regimen resulted in intermediate antitumor efficacy, superior to ADC monotherapy and [^177^Lu]Lu-labeled Ab treatment, but consistently inferior to simultaneous [^177^Lu]Lu-B7-H3 ADC administration. These findings indicate that temporal separation of ADC and radionuclide delivery does not fully provide the therapeutic benefit observed with the integrated construct. Rather than reflecting a simple additive effect of sequential cytotoxic insults, the superior efficacy of [^177^Lu]Lu-B7-H3 ADC appears to depend on concurrent delivery and spatial co-localization of radionuclide and payload at the tumor site, reinforcing the concept that molecular integration is a defining feature of effective theranostic design.

Mechanistically, this integrated approach likely benefits from complementary modes of action. Targeted β-emission from [^177^Lu]Lu induces DNA damage within tumor cells and the surrounding microenvironment 37, while the ADC delivers cytotoxic payloads selectively to B7-H3-expressing cells 14. Radiation-induced immunogenic cell death has been reported to promote antitumor immune responses through the release of tumor-associated antigens and damage-associated molecular patterns 38, 39. However, such immune-mediated mechanisms cannot be functionally evaluated in the present study due to the immunodeficient background of the mouse model used. Importantly, B7-H3 expression remained consistently high across all treatment groups post-therapy. This persistence of target expression can be attributed to the mechanism of B7-H3-targeted ADC agents, which inhibit the downstream signaling pathways rather downregulating the expression of B7-H3 40, 41. This characteristic significantly reduces the likelihood of tumors developing resistance to [^177^Lu]Lu-B7-H3 ADC therapy, representing a substantial clinical advantage.

From a safety perspective, [^177^Lu]Lu-B7-H3 ADC demonstrated a manageable toxicity profile. Histopathological and biochemical analyses revealed no additional organ toxicity attributable to radionuclide conjugation when compared with ADC monotherapy. Transient hematological changes were observed, consistent with known ADC-associated effects, but recovered over time. These findings suggest that incorporation of [^177^Lu]Lu enhances therapeutic efficacy without fundamentally altering the safety boundary of the ADC platform. Future development efforts could focus on optimizing the probe structure to accelerate blood clearance while maintaining tumor uptake, potentially through engineering smaller proteins 42.

Notably, the B7-H3 ADC used in this study does not cross-react with murine B7-H3. Therefore, safety findings in healthy BALB/c mice primarily reflect non-specific pharmacokinetics and radiation-related effects rather than target-mediated uptake in normal tissues, which should be considered when extrapolating toxicity profiles across species. In humans, B7-H3 is generally reported to show limited expression in normal tissues compared with tumors, supporting a potentially favorable therapeutic window 11-13.

Several limitations warrant consideration. First, 22RV1 models exhibits moderate B7-H3 expression, it was selected based on its reproducible tumor growth characteristics and experimental robustness. Evaluation of additional models spanning a broader B7-H3 expression spectrum (such as LNCaP and PC3) may further refine PET-based stratification. Second, while single-dose administration demonstrated strong efficacy in preclinical models, optimization of dosing schedules and fractionation strategies will be necessary for clinical translation. Third, the single administration targeting strategy may face challenges in clinically heterogeneous tumor environments. This limitation could potentially be overcome through the development of bispecific constructs such as B7-H3 and PSMA 43, PSMA and six-transmembrane epithelial antigen of the prostate 1 (STEAP1) 44.

## Conclusions

In conclusion, this study establishes a B7-H3-targeted [^89^Zr]Zr- / [^177^Lu]Lu-ADC theranostic platform that enables precise tumor targeting, effective immunoPET imaging, and potent antitumor activity in prostate cancer models. By integrating radionuclide therapy with ADC-based targeting, this strategy achieves enhanced therapeutic efficacy at reduced ADC doses without increasing systemic toxicity. These findings support the clinical translation of [^177^Lu]Lu-B7-H3 ADC as a promising and truly synergistic theranostic approach for patients with B7-H3-expressing prostate cancer.

## Supplementary Material

Supplementary figures.

## Data Availability

Data will be available upon reasonable request to Lei Kang (kanglei@bjmu.edu.cn).

## Abbreviations

Ab: antibody
ADC: antibody-drug conjugate
ALT: alanine aminotransferase
ANOVA: analysis of variance
AST: aspartate transaminase
B7-H3: B7 homolog 3
CREA: creatinine
CT: computed tomography
DFO: desferrioxamine
DOTA: 1,4,7,10-tetraazacyclododecane-1,4,7,10-tetraacetic acid
FBS: fetal bovine serum
H&E: hematoxylin and eosin
HGB: hemoglobin
IHC: immunohistochemistry
IQR: interquartile range
ISUP: International Society of Urological Pathology
MIP: maximum intensity projection
PBS: phosphate-buffered saline
PET: positron emission tomography
PSMA: prostate-specific membrane antigen
RBC: red blood cell
ROI: region of interest
SD: standard deviation
SPECT: single-photon emission computed tomography
STEAP1: six-transmembrane epithelial antigen of the prostate 1
TBR: tumor-to-background ratio
TLC: thin-layer chromatography
WB: western blot
WBC: white blood cell
