# Supplementary material for: Integrated radio-theranostics using a [89Zr]Zr-/[177Lu]Lu-labeled B7-H3 antibody-drug conjugate for prostate cancer
Source: Theranostics. 2026 Apr 16;16(11):6132–44. doi: 10.7150/thno.125878 (PMC13142242; doi:10.7150/thno.125878)
Supplement: Supplementary file 1 — Supplementary figures. [file thnov16p6132s1.pdf]

Supplementary material

Table S1. Baseline information of prostate cancer patients

	All patients (N = 40)	Primary tumor (N = 20)	Bone metastasis (N = 20)
Age	66.5 (64.8–73.3)	65.0 (63.8–71.0)	69.0 (66.0–74.2)
t-PSA (ng/ml)	23.4 (12.7–79.4)	15.8 (8.9–23.6)	85.3 (24.0–495.3)
Primary tumor ISUP			
ISUP = 1		1 (5%)	
ISUP = 2		7 (35%)	
ISUP = 3		3 (15%)	
ISUP = 4		1 (5%)	
ISUP = 5		8 (40%)	
Bone metastasis's AR expression			
Negative			4 (20%)
Weak			2 (10%)
Moderate			6 (30%)
Strong			8 (40%)

ISUP: International Society of Urological Pathology

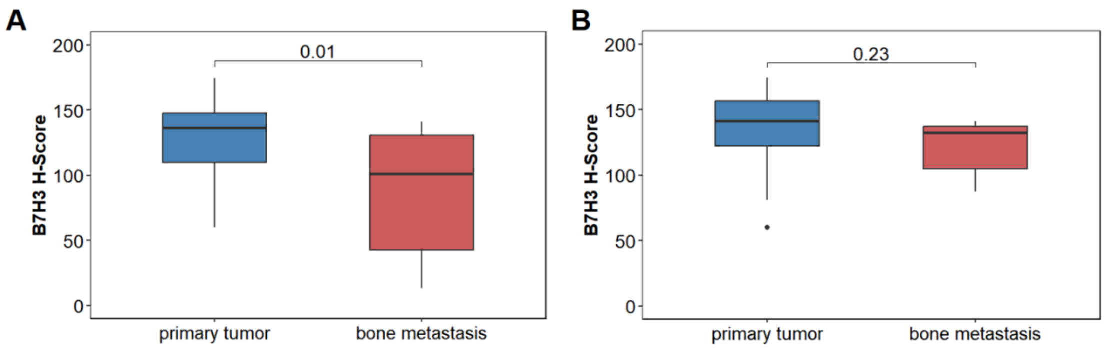

Figure S1. Quantitative comparison of H-scores for B7-H3 expression in primary tumors and bone metastases (A) and immunohistochemistry staining strong samples (B).

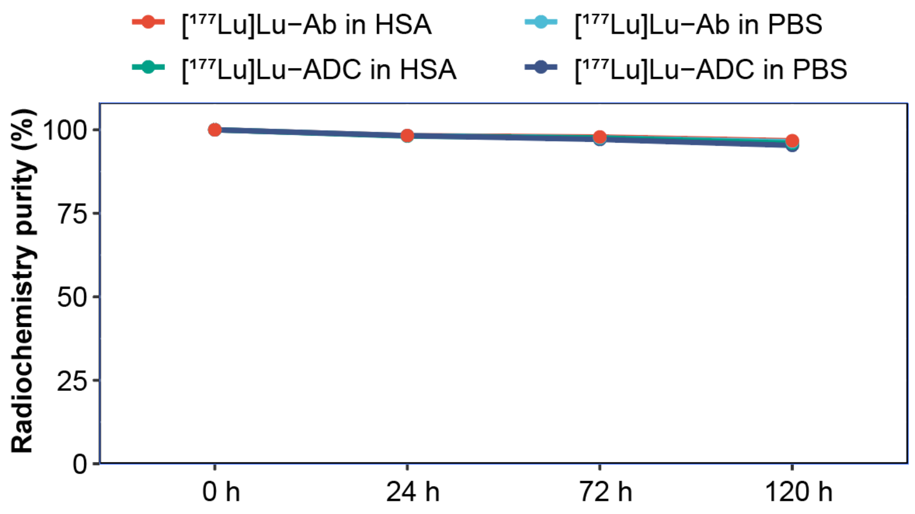

Figure S2. Radiochemical purity of [^{177}Lu]Lu-B7-H3 Ab and [^{177}Lu]Lu-B7-H3 ADC in human serum albumin (HSA) and Phosphate Buffered Saline (PBS).

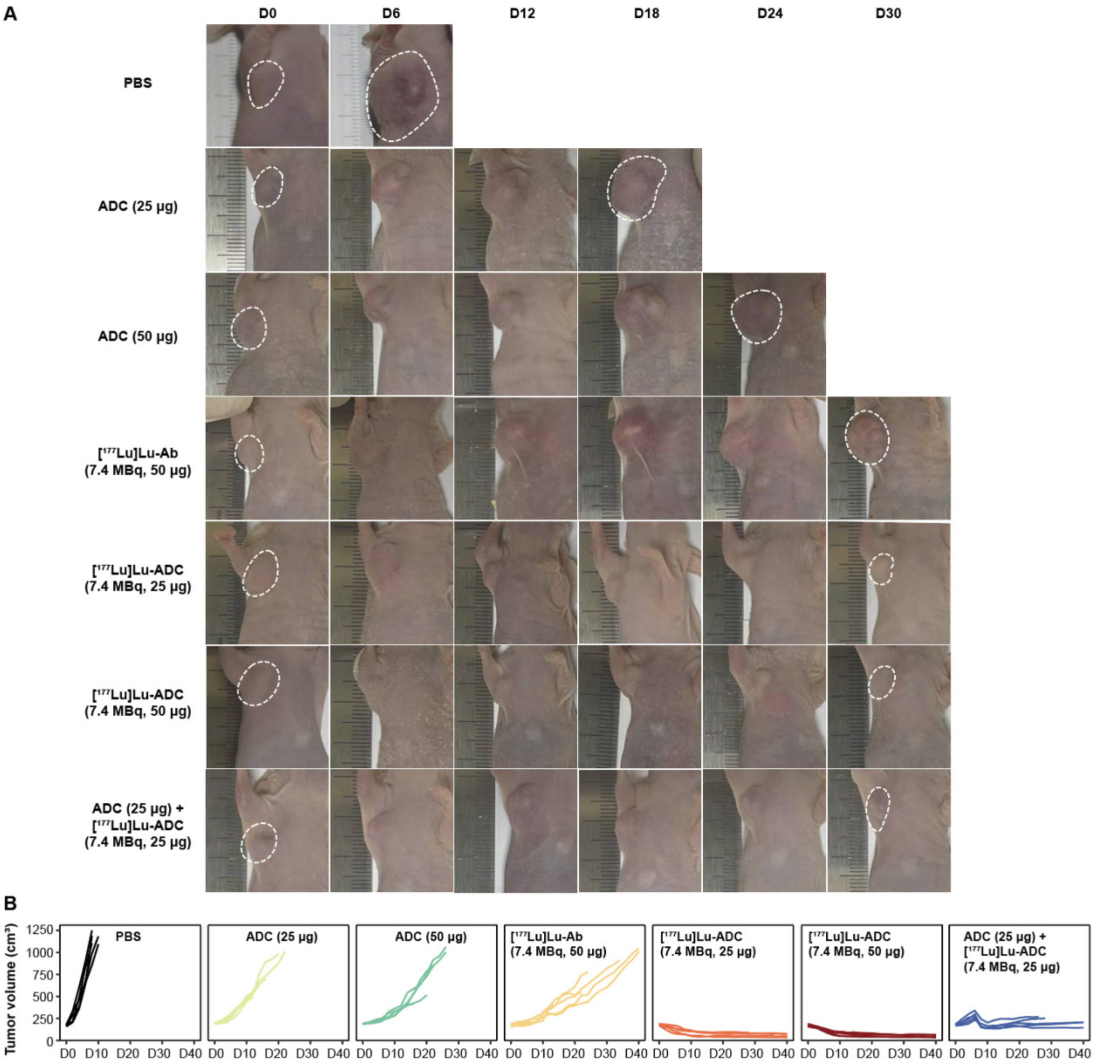

Figure S3. Therapeutic efficacy and safety evaluation of [^{177}Lu]Lu-B7-H3 ADC (1.9 MBq, 50 µg) in 22RV1 tumor-bearing mice.

A. Representative photographs of tumor-bearing mice. Overall therapeutic efficacy followed the order: [^{177}Lu]Lu-ADC (7.4 MBq, 50 µg) ≈ [^{177}Lu]Lu-ADC (7.4 MBq, 25 µg) > sequential therapy > [^{177}Lu]Lu-Ab (7.4 MBq, 50 µg) > ADC (50 µg) > ADC (25 µg) > PBS.

B. Individual tumor growth trajectories further illustrated the consistency of therapeutic responses (n = 5).

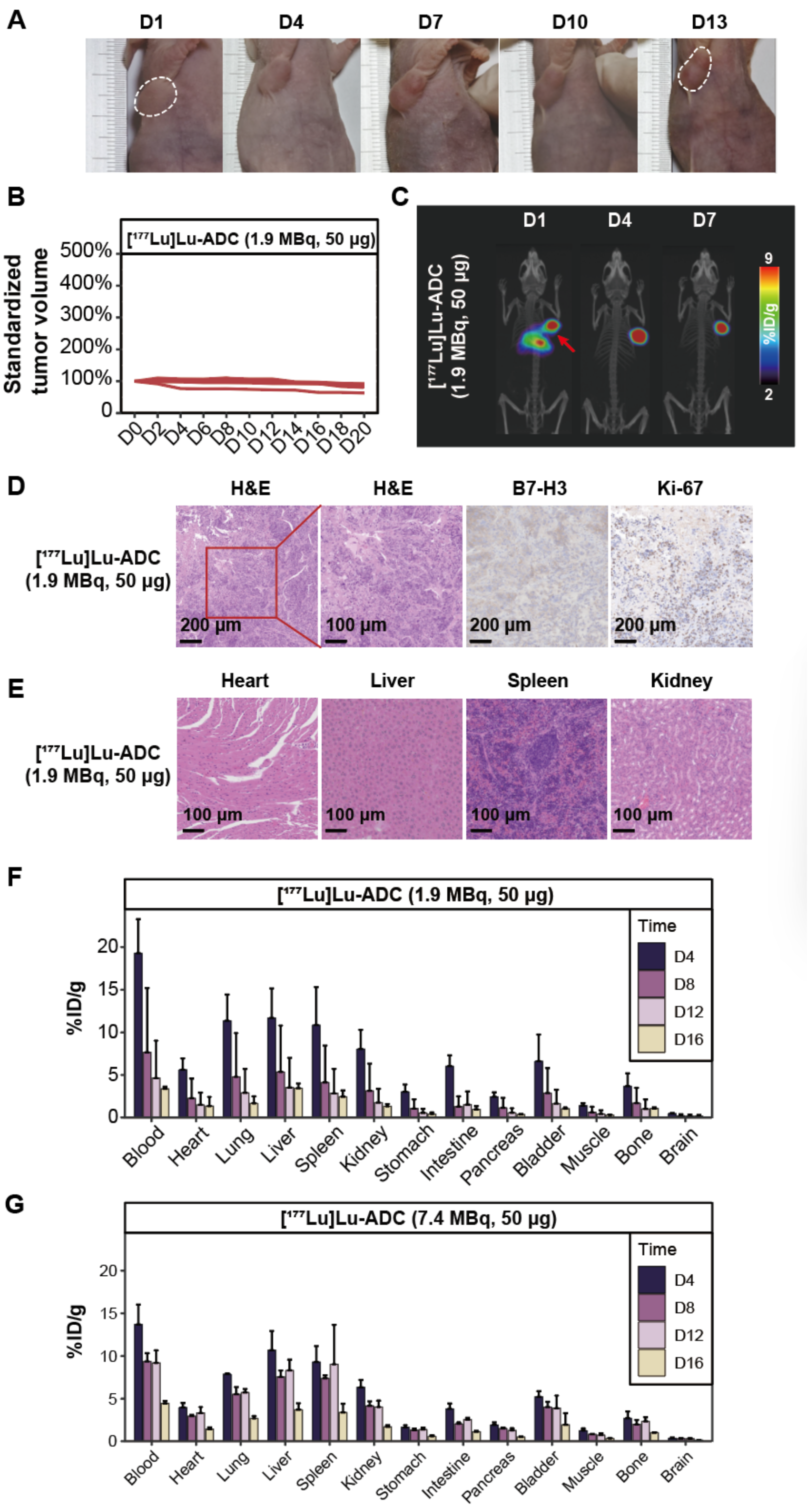

Figure S4. Therapeutic efficacy and safety evaluation of [^{177}Lu]Lu-B7-H3 ADC (1.9 MBq, 50 μg) in 22RV1 tumor-bearing mice.

A. Representative tumor region photographs of [^{177}Lu]Lu-B7-H3 ADC (1.9 MBq, 50 μg) in 22RV1 tumor-bearing mice.

B. Individual tumor growth trajectories of [^{177}Lu]Lu-B7-H3 ADC (1.9 MBq, 50 μg) in 22RV1

tumor-bearing mice (n = 5).

C. Serial SPECT/CT maximum intensity projection (MIP) images of [^{177}Lu]Lu-B7-H3 ADC (1.9 MBq, 50 μg) in 22RV1 tumor-bearing mice at D1, D4, D7 post injection (red arrows indicate tumor foci).

D. Representative H&E staining, B7-H3 immunohistochemistry and Ki-67 staining of [^{177}Lu]Lu-B7-H3 ADC (1.9 MBq, 50 μg) in 22RV1 tumor-bearing mice.

E. Representative H&E staining sections of major organs (heart, liver, spleen and kidney) at D20 post-injection.

F. Biodistribution analysis of [^{177}Lu]Lu-ADC (1.9 MBq, 50μg).

G. Biodistribution analysis of [^{177}Lu]Lu-ADC (7.4 MBq, 50μg).

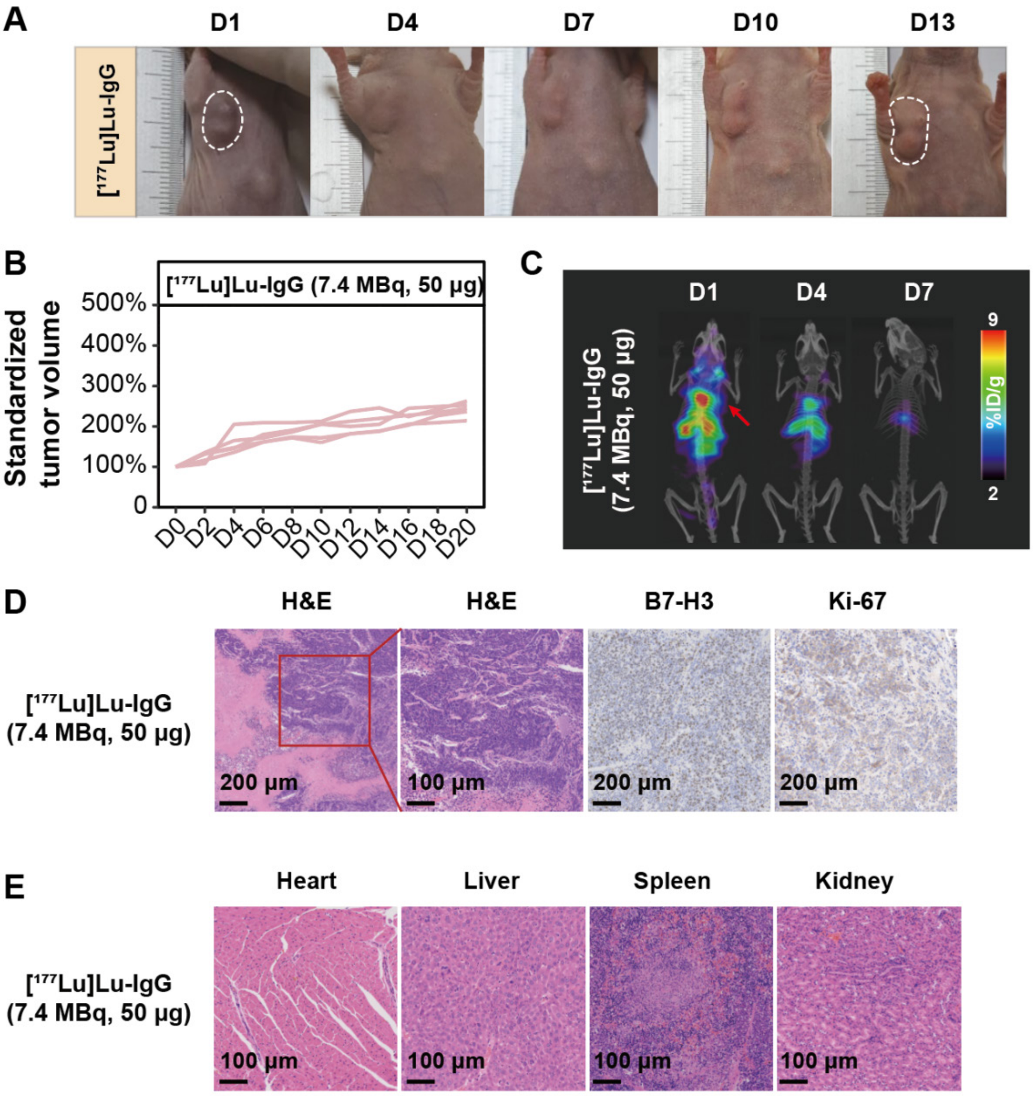

Figure S5. Therapeutic efficacy and safety evaluation of [^{177}Lu]Lu-IgG (7.4 MBq, 50 μg) in 22RV1 tumor-bearing mice.

A. Representative tumor region photographs of [^{177}Lu]Lu-IgG (7.4 MBq, 50 μg) in 22RV1 tumor-bearing mice.
B. Individual tumor growth trajectories of [^{177}Lu]Lu-IgG (7.4 MBq, 50 μg) in 22RV1 tumor-bearing mice (n = 5).
C. Serial SPECT/CT maximum intensity projection (MIP) images of [^{177}Lu]Lu-IgG (7.4 MBq, 50 μg) in 22RV1 tumor-bearing mice at D1, D4, D7 post injection (red arrows indicate tumor foci).
D. Representative H&E staining, B7-H3 immunohistochemistry and Ki-67 staining of [^{177}Lu]Lu-IgG (7.4 MBq, 50 μg) in 22RV1 tumor-bearing mice.
E. Representative H&E staining sections of major organs (heart, liver, spleen and kidney) at D20 post-injection.

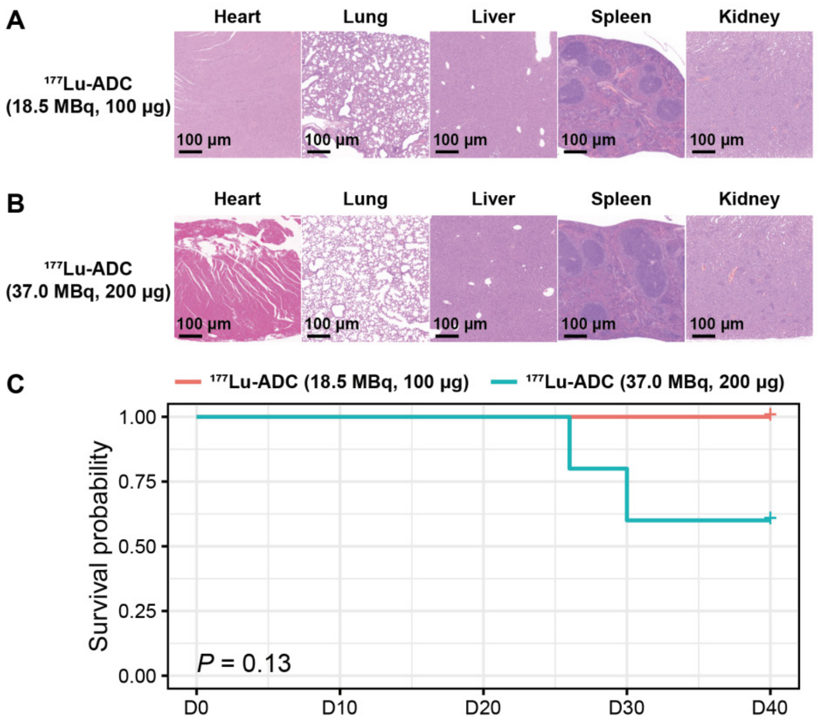

Figure S6. Safety evaluation of naximum tolerated [^{177}Lu]Lu-B7-H3 ADC in 22RV1 tumor-bearing mice.

A. Representative H&E staining sections of major organs (heart, lung, liver, spleen and kidney) at D20 post-injection in [^{177}Lu]Lu-B7-H3 ADC (18.5 MBq, 100 μg).

B. Representative H&E staining sections of major organs (heart, lung, liver, spleen and kidney) at D20 post-injection in [^{177}Lu]Lu-B7-H3 ADC (37.0 MBq, 200 μg).

C. Kaplan–Meier survival analysis demonstrated the survival outcome of mice treated with [^{177}Lu]Lu-B7-H3 ADC (18.5 MBq, 100 μg) and [^{177}Lu]Lu-B7-H3 ADC (37.0 MBq, 200 μg) (n = 5).
